# 3-Phenyl-2-(piperidin-1-yl)-3,5,6,8-tetra­hydro-4*H*-thio­pyrano[3′,4′:2,3]thieno[5,4-*d*]pyrimidin-4-one

**DOI:** 10.1107/S1600536808038683

**Published:** 2008-11-26

**Authors:** Hai Xie, Shuang-Ming Meng, Yue-Qin Fan, Yong Guo

**Affiliations:** a College of Chemistry and Chemical Engineering, ShanXi Datong University, Datong, Shanxi 037009, People’s Republic of China

## Abstract

In the title compound, C_20_H_21_N_3_OS_2_, the piperidinyl ring has a distorted chair conformation. Weak inter­molecular C—H⋯O hydrogen bonds link the mol­ecules into centrosymmetric dimers. The crystal packing exhibits short inter­molecular S⋯S distances of 3.590 (2) Å.

## Related literature

For properties of the compounds containing th thienopyrimidine system, see: Muller *et al.* (2002 ▶); Chambhare *et al.* (2003 ▶). For related crystal structures, see: Hu *et al.* (2007 ▶); Xie *et al.* (2007 ▶).
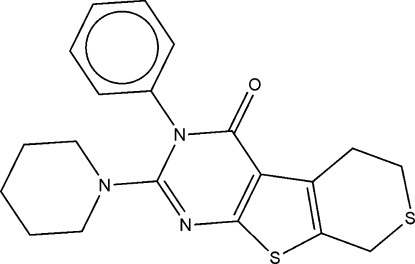


## Experimental

### 

#### Crystal data

C_20_H_21_N_3_OS_2_
*M* *_r_* = 383.52
Triclinic, 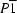

*a* = 9.851 (2) Å
*b* = 10.755 (3) Å
*c* = 10.864 (3) Å
α = 117.573 (4)°
β = 106.099 (4)°
γ = 97.322 (4)°
*V* = 935.0 (4) Å^3^
*Z* = 2
Mo *K*α radiation
μ = 0.30 mm^−1^
*T* = 298 (2) K
0.26 × 0.12 × 0.06 mm

#### Data collection

Bruker SMART CCD area-detector diffractometer
Absorption correction: multi-scan (*SADABS*; Sheldrick, 1996 ▶) *T*_min_ = 0.926, *T*_max_ = 0.982
4908 measured reflections
3203 independent reflections
2739 reflections with *I* > 2σ(*I*)
*R*_int_ = 0.020

#### Refinement

*R*[*F* ^2^ > 2σ(*F* ^2^)] = 0.055
*wR*(*F* ^2^) = 0.146
*S* = 1.08
3203 reflections
235 parameters
H-atom parameters constrained
Δρ_max_ = 0.29 e Å^−3^
Δρ_min_ = −0.35 e Å^−3^

### 

Data collection: *SMART* (Bruker, 2007 ▶); cell refinement: *SAINT* (Bruker, 2007 ▶); data reduction: *SAINT*; program(s) used to solve structure: *SHELXS97* (Sheldrick, 2008 ▶); program(s) used to refine structure: *SHELXL97* (Sheldrick, 2008 ▶); molecular graphics: *SHELXTL* (Sheldrick, 2008 ▶); software used to prepare material for publication: *SHELXTL*.

## Supplementary Material

Crystal structure: contains datablocks global, I. DOI: 10.1107/S1600536808038683/cv2480sup1.cif

Structure factors: contains datablocks I. DOI: 10.1107/S1600536808038683/cv2480Isup2.hkl

Additional supplementary materials:  crystallographic information; 3D view; checkCIF report

## Figures and Tables

**Table 1:** Hydrogen-bond geometry (Å, °)

*D*—H⋯*A*	*D*—H	H⋯*A*	*D*⋯*A*	*D*—H⋯*A*
C7—H7*B*⋯O1^i^	0.97	2.56	3.321 (5)	136

Symmetry code: (i) 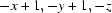.

**Fig. 1.**
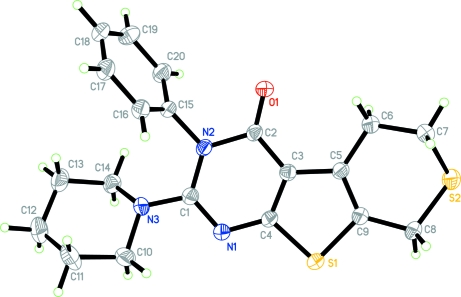
The molecular structure of (I) with displacement ellipsoids drawn at the 30% probability level.

**Fig. 2.**
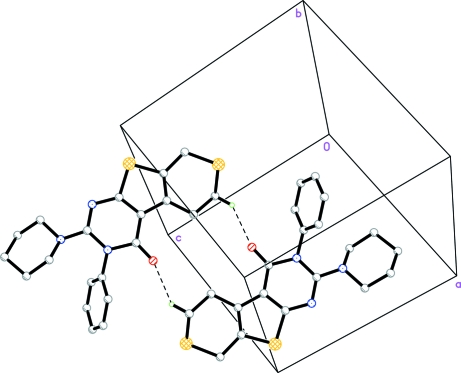
A portion of the crystal packing showing hydrogen bonds as dashed lines. H atoms, except for those involved in hydrogen bonds, are not included.

## supplementary crystallographic information

### Comment

The derivatives of heterocycles containing the thienopyrimidine system have proved to show significant antifungal, antibacterical, anticonvulsant and angiotensin antagonistic activities(Muller *et al.*,2002; Chambhare *et al.*2003). Recently, we have focused on the synthesis of fused heterocyclic systems containing thienopyrimidine via aza-wittig reaction at room temperature. Some X-ray crystal structures of fused pyrimidinone derivatives have been reported (Xie *et al.*, 2007; Hu *et al.*, 2007). The title compound (I) can be used as a new precursor for obtaining of bioactive molecules with fluorescence properties.

In (I) (Fig. 1), the piperidinyl ring has a distored chair conformation. The weak intermolecular C—H···O hydrogen bonds (Table 2) link the molecules into centrosymmetric dimers (Fig. 2). The crystal packing exhibits relatively short intermolecular S···S distances of 3.590 (2) Å (Table 1), which is shorter than the sum of the van der Waals radii of the relevent atoms.

### Experimental

To a solution of iminophosphorane(2mmol) in anhyd.CH_2_Cl_2_(10ml)aromatic isocyanate(2mmol)was added under nitrogen atmosphere at room temperature.After the reaction mixture was left unstirred for 6-12h at 0-5°C,the iminophosphorane had disappeared (TLC monitored).The solvent was removed off under redunced pressure and Et_2_O/petroleum ether (1:2,20ml) was added to precipitate triphenylphosphine oxide. Removal of the solvent gave carbodiimides,which were used directly without further purification. To a solution of carbodiimides in CH_2_Cl_2_(10ml)dialkylamine(2mmol). After the reaction mixture was left unstirred for 4-6h. The solvent was removed and anhyd.EtOH(10ml)with several drops of EtONa in EtOH was added. The mixture was stirred for 6-12h at room temperature. The solution was condensed and residue was recrystallized from EtOH to give the expected title compound in a good yield.

### Refinement

All H atoms were positioned geometrically [C-H=0.93, 0.97 Å] and allowed to ride on their parent atoms ,  with U_iso_(H)=1.2Ueq(C).

### Crystal data

**Table:**

C_20_H_21_N_3_OS_2_	*Z* = 2
*M**_r_* = 383.52	*F*_000_ = 404
Triclinic, *P*1	*D*_x_ = 1.362 Mg m^−^^3^
Hall symbol: -P 1	Mo *K*α radiation λ = 0.71073 Å
*a* = 9.851 (2) Å	Cell parameters from 2241 reflections
*b* = 10.755 (3) Å	θ = 2.2–27.6º
*c* = 10.864 (3) Å	µ = 0.30 mm^−^^1^
α = 117.573 (4)º	*T* = 298 (2) K
β = 106.099 (4)º	Block, red
γ = 97.322 (4)º	0.26 × 0.12 × 0.06 mm
*V* = 935.0 (4) Å^3^	

### Data collection

**Table:**

Bruker SMART CCD area-detector diffractometer	3203 independent reflections
Radiation source: fine-focus sealed tube	2739 reflections with *I* > 2σ(*I*)
Monochromator: graphite	*R*_int_ = 0.020
Detector resolution: 0 pixels mm^-1^	θ_max_ = 25.0º
*T* = 298(2) K	θ_min_ = 2.2º
φ and ω scans	*h* = −11→11
Absorption correction: multi-scan(SADABS; Sheldrick, 1996)	*k* = −6→12
*T*_min_ = 0.926, *T*_max_ = 0.982	*l* = −12→12
4908 measured reflections	

### Refinement

**Table:**

Refinement on *F*^2^	Secondary atom site location: difference Fourier map
Least-squares matrix: full	Hydrogen site location: inferred from neighbouring sites
*R*[*F*^2^ > 2σ(*F*^2^)] = 0.055	H-atom parameters constrained
*wR*(*F*^2^) = 0.146	*w* = 1/[σ^2^(*F*_o_^2^) + (0.0458*P*)^2^ + 1.1731*P*] where *P* = (*F*_o_^2^ + 2*F*_c_^2^)/3
*S* = 1.08	(Δ/σ)_max_ < 0.001
3203 reflections	Δρ_max_ = 0.29 e Å^−^^3^
235 parameters	Δρ_min_ = −0.35 e Å^−^^3^
Primary atom site location: structure-invariant direct methods	Extinction correction: none

### Special details

**Table:**

Geometry. All esds (except the esd in the dihedral angle between two l.s. planes) are estimated using the full covariance matrix. The cell esds are taken into account individually in the estimation of esds in distances, angles and torsion angles; correlations between esds in cell parameters are only used when they are defined by crystal symmetry. An approximate (isotropic) treatment of cell esds is used for estimating esds involving l.s. planes.
Refinement. Refinement of F^2^ against ALL reflections. The weighted R-factor wR and goodness of fit S are based on F^2^, conventional R-factors R are based on F, with F set to zero for negative F^2^. The threshold expression of F^2^ > 2sigma(F^2^) is used only for calculating R-factors(gt) etc. and is not relevant to the choice of reflections for refinement. R-factors based on F^2^ are statistically about twice as large as those based on F, and R- factors based on ALL data will be even larger.

### Fractional atomic coordinates and isotropic or equivalent isotropic displacement parameters (Å^2^)

**Table:**

	*x*	*y*	*z*	*U*_iso_*/*U*_eq_	
O1	0.4581 (3)	0.6122 (3)	0.2373 (3)	0.0512 (6)	
S1	0.01324 (10)	0.73341 (11)	0.03936 (9)	0.0504 (3)	
S2	0.11865 (11)	0.48110 (13)	−0.35508 (10)	0.0600 (3)	
N1	0.1593 (3)	0.8366 (3)	0.3342 (3)	0.0427 (6)	
N2	0.3694 (3)	0.7625 (3)	0.4072 (3)	0.0365 (6)	
N3	0.2786 (3)	0.9126 (3)	0.5845 (3)	0.0425 (6)	
C1	0.2676 (3)	0.8387 (3)	0.4386 (3)	0.0376 (7)	
C2	0.3656 (3)	0.6767 (3)	0.2579 (3)	0.0355 (7)	
C3	0.2474 (3)	0.6762 (3)	0.1466 (3)	0.0365 (7)	
C4	0.1518 (3)	0.7530 (3)	0.1913 (3)	0.0392 (7)	
C5	0.2090 (3)	0.5983 (3)	−0.0135 (3)	0.0383 (7)	
C6	0.2920 (4)	0.5025 (4)	−0.0928 (4)	0.0468 (8)	
H6A	0.3231	0.4483	−0.0449	0.056*	
H6B	0.3803	0.5643	−0.0824	0.056*	
C7	0.2004 (4)	0.3946 (4)	−0.2580 (4)	0.0543 (9)	
H7A	0.1223	0.3216	−0.2682	0.065*	
H7B	0.2629	0.3440	−0.3053	0.065*	
C8	0.0051 (4)	0.5529 (4)	−0.2501 (4)	0.0485 (8)	
H8A	−0.0263	0.6272	−0.2677	0.058*	
H8B	−0.0830	0.4741	−0.2862	0.058*	
C9	0.0850 (3)	0.6190 (4)	−0.0851 (3)	0.0417 (7)	
C10	0.1465 (4)	0.9449 (5)	0.6117 (4)	0.0597 (10)	
H10A	0.0584	0.8670	0.5301	0.072*	
H10B	0.1404	1.0364	0.6168	0.072*	
C11	0.1558 (5)	0.9573 (5)	0.7584 (5)	0.0766 (13)	
H11A	0.0709	0.9837	0.7797	0.092*	
H11B	0.1519	0.8624	0.7485	0.092*	
C12	0.2958 (5)	1.0705 (5)	0.8881 (4)	0.0795 (14)	
H12A	0.3021	1.0675	0.9774	0.095*	
H12B	0.2931	1.1679	0.9086	0.095*	
C13	0.4306 (5)	1.0415 (5)	0.8524 (4)	0.0651 (11)	
H13A	0.5185	1.1213	0.9317	0.078*	
H13B	0.4420	0.9512	0.8475	0.078*	
C14	0.4149 (4)	1.0284 (4)	0.7049 (4)	0.0490 (8)	
H14A	0.4121	1.1215	0.7127	0.059*	
H14B	0.4998	1.0054	0.6812	0.059*	
C15	0.4696 (3)	0.7434 (3)	0.5194 (3)	0.0385 (7)	
C16	0.4120 (4)	0.6656 (4)	0.5735 (4)	0.0446 (8)	
H16A	0.3100	0.6287	0.5416	0.053*	
C17	0.5068 (5)	0.6429 (4)	0.6754 (4)	0.0562 (10)	
H17A	0.4684	0.5906	0.7127	0.067*	
C18	0.6570 (5)	0.6964 (5)	0.7224 (4)	0.0635 (11)	
H18A	0.7202	0.6793	0.7904	0.076*	
C19	0.7144 (4)	0.7753 (5)	0.6691 (4)	0.0643 (11)	
H19A	0.8165	0.8127	0.7021	0.077*	
C20	0.6205 (4)	0.7991 (4)	0.5661 (4)	0.0515 (9)	
H20A	0.6588	0.8519	0.5291	0.062*	

### Atomic displacement parameters (Å^2^)

**Table:**

	*U*^11^	*U*^22^	*U*^33^	*U*^12^	*U*^13^	*U*^23^
O1	0.0471 (14)	0.0719 (16)	0.0425 (13)	0.0345 (13)	0.0203 (11)	0.0300 (12)
S1	0.0461 (5)	0.0677 (6)	0.0376 (5)	0.0310 (4)	0.0126 (4)	0.0261 (4)
S2	0.0618 (6)	0.0872 (8)	0.0411 (5)	0.0310 (5)	0.0237 (4)	0.0367 (5)
N1	0.0430 (15)	0.0485 (16)	0.0358 (14)	0.0220 (13)	0.0143 (12)	0.0199 (13)
N2	0.0329 (13)	0.0464 (15)	0.0336 (13)	0.0116 (12)	0.0110 (11)	0.0244 (12)
N3	0.0432 (15)	0.0450 (16)	0.0313 (13)	0.0145 (13)	0.0140 (12)	0.0137 (12)
C1	0.0368 (16)	0.0380 (17)	0.0366 (16)	0.0102 (14)	0.0130 (13)	0.0193 (14)
C2	0.0355 (16)	0.0433 (17)	0.0346 (16)	0.0123 (14)	0.0131 (13)	0.0256 (14)
C3	0.0319 (16)	0.0394 (17)	0.0366 (16)	0.0078 (13)	0.0124 (13)	0.0196 (14)
C4	0.0403 (17)	0.0434 (18)	0.0362 (16)	0.0170 (15)	0.0115 (14)	0.0233 (14)
C5	0.0342 (16)	0.0437 (18)	0.0369 (16)	0.0110 (14)	0.0121 (13)	0.0219 (14)
C6	0.0426 (18)	0.056 (2)	0.0397 (18)	0.0184 (16)	0.0161 (15)	0.0229 (16)
C7	0.052 (2)	0.065 (2)	0.0411 (19)	0.0272 (19)	0.0196 (16)	0.0212 (18)
C8	0.0454 (19)	0.064 (2)	0.0355 (17)	0.0203 (17)	0.0105 (15)	0.0270 (17)
C9	0.0394 (17)	0.0490 (19)	0.0356 (17)	0.0146 (15)	0.0129 (14)	0.0217 (15)
C10	0.050 (2)	0.063 (2)	0.049 (2)	0.0232 (19)	0.0186 (17)	0.0146 (18)
C11	0.084 (3)	0.083 (3)	0.061 (3)	0.025 (3)	0.048 (2)	0.026 (2)
C12	0.101 (4)	0.087 (3)	0.040 (2)	0.035 (3)	0.031 (2)	0.021 (2)
C13	0.073 (3)	0.064 (3)	0.0358 (19)	0.020 (2)	0.0086 (18)	0.0164 (18)
C14	0.052 (2)	0.0406 (19)	0.0397 (18)	0.0122 (16)	0.0104 (16)	0.0147 (15)
C15	0.0372 (17)	0.0429 (18)	0.0314 (15)	0.0153 (14)	0.0097 (13)	0.0176 (14)
C16	0.0477 (19)	0.0467 (19)	0.0401 (18)	0.0159 (16)	0.0159 (15)	0.0234 (16)
C17	0.078 (3)	0.064 (2)	0.044 (2)	0.037 (2)	0.0265 (19)	0.0366 (19)
C18	0.072 (3)	0.084 (3)	0.041 (2)	0.047 (2)	0.0161 (19)	0.034 (2)
C19	0.038 (2)	0.085 (3)	0.056 (2)	0.021 (2)	0.0055 (17)	0.033 (2)
C20	0.0413 (19)	0.065 (2)	0.048 (2)	0.0136 (17)	0.0137 (16)	0.0320 (18)

### Geometric parameters (Å, °)

**Table:**

O1—C2	1.221 (4)	C10—C11	1.512 (6)
S1—C4	1.729 (3)	C10—H10A	0.9700
S1—C9	1.748 (3)	C10—H10B	0.9700
S2—C7	1.804 (4)	C11—C12	1.508 (6)
S2—C8	1.806 (3)	C11—H11A	0.9700
N1—C1	1.313 (4)	C11—H11B	0.9700
N1—C4	1.361 (4)	C12—C13	1.514 (6)
N2—C1	1.390 (4)	C12—H12A	0.9700
N2—C2	1.433 (4)	C12—H12B	0.9700
N2—C15	1.456 (4)	C13—C14	1.503 (5)
N3—C1	1.369 (4)	C13—H13A	0.9700
N3—C10	1.462 (4)	C13—H13B	0.9700
N3—C14	1.468 (4)	C14—H14A	0.9700
C2—C3	1.426 (4)	C14—H14B	0.9700
C3—C4	1.372 (4)	C15—C16	1.374 (5)
C3—C5	1.440 (4)	C15—C20	1.379 (5)
C5—C9	1.360 (4)	C16—C17	1.375 (5)
C5—C6	1.502 (4)	C16—H16A	0.9300
C6—C7	1.513 (5)	C17—C18	1.369 (6)
C6—H6A	0.9700	C17—H17A	0.9300
C6—H6B	0.9700	C18—C19	1.375 (6)
C7—H7A	0.9700	C18—H18A	0.9300
C7—H7B	0.9700	C19—C20	1.388 (5)
C8—C9	1.495 (4)	C19—H19A	0.9300
C8—H8A	0.9700	C20—H20A	0.9300
C8—H8B	0.9700		
			
S2···S2^i^	3.590 (2)		
			
C4—S1—C9	91.31 (15)	C11—C10—H10A	109.9
C7—S2—C8	97.82 (16)	N3—C10—H10B	109.9
C1—N1—C4	115.4 (3)	C11—C10—H10B	109.9
C1—N2—C2	122.6 (2)	H10A—C10—H10B	108.3
C1—N2—C15	121.4 (2)	C12—C11—C10	112.3 (4)
C2—N2—C15	115.1 (2)	C12—C11—H11A	109.1
C1—N3—C10	117.7 (3)	C10—C11—H11A	109.1
C1—N3—C14	120.9 (3)	C12—C11—H11B	109.1
C10—N3—C14	111.8 (3)	C10—C11—H11B	109.1
N1—C1—N3	119.5 (3)	H11A—C11—H11B	107.9
N1—C1—N2	122.9 (3)	C11—C12—C13	110.6 (3)
N3—C1—N2	117.5 (3)	C11—C12—H12A	109.5
O1—C2—C3	126.9 (3)	C13—C12—H12A	109.5
O1—C2—N2	119.6 (3)	C11—C12—H12B	109.5
C3—C2—N2	113.5 (3)	C13—C12—H12B	109.5
C4—C3—C2	118.4 (3)	H12A—C12—H12B	108.1
C4—C3—C5	113.6 (3)	C14—C13—C12	110.4 (3)
C2—C3—C5	127.9 (3)	C14—C13—H13A	109.6
N1—C4—C3	127.1 (3)	C12—C13—H13A	109.6
N1—C4—S1	121.6 (2)	C14—C13—H13B	109.6
C3—C4—S1	111.2 (2)	C12—C13—H13B	109.6
C9—C5—C3	111.4 (3)	H13A—C13—H13B	108.1
C9—C5—C6	123.9 (3)	N3—C14—C13	110.4 (3)
C3—C5—C6	124.7 (3)	N3—C14—H14A	109.6
C5—C6—C7	112.8 (3)	C13—C14—H14A	109.6
C5—C6—H6A	109.0	N3—C14—H14B	109.6
C7—C6—H6A	109.0	C13—C14—H14B	109.6
C5—C6—H6B	109.0	H14A—C14—H14B	108.1
C7—C6—H6B	109.0	C16—C15—C20	121.0 (3)
H6A—C6—H6B	107.8	C16—C15—N2	119.3 (3)
C6—C7—S2	113.1 (3)	C20—C15—N2	119.7 (3)
C6—C7—H7A	109.0	C15—C16—C17	119.3 (3)
S2—C7—H7A	109.0	C15—C16—H16A	120.4
C6—C7—H7B	109.0	C17—C16—H16A	120.4
S2—C7—H7B	109.0	C18—C17—C16	120.7 (4)
H7A—C7—H7B	107.8	C18—C17—H17A	119.6
C9—C8—S2	112.4 (2)	C16—C17—H17A	119.6
C9—C8—H8A	109.1	C17—C18—C19	119.9 (3)
S2—C8—H8A	109.1	C17—C18—H18A	120.1
C9—C8—H8B	109.1	C19—C18—H18A	120.1
S2—C8—H8B	109.1	C18—C19—C20	120.2 (4)
H8A—C8—H8B	107.9	C18—C19—H19A	119.9
C5—C9—C8	128.4 (3)	C20—C19—H19A	119.9
C5—C9—S1	112.4 (2)	C15—C20—C19	118.9 (4)
C8—C9—S1	119.1 (2)	C15—C20—H20A	120.5
N3—C10—C11	108.7 (3)	C19—C20—H20A	120.5
N3—C10—H10A	109.9		
			
C4—N1—C1—N3	−176.1 (3)	C5—C6—C7—S2	52.2 (4)
C4—N1—C1—N2	0.6 (5)	C8—S2—C7—C6	−61.9 (3)
C10—N3—C1—N1	19.5 (5)	C7—S2—C8—C9	42.6 (3)
C14—N3—C1—N1	−124.1 (3)	C3—C5—C9—C8	−177.1 (3)
C10—N3—C1—N2	−157.4 (3)	C6—C5—C9—C8	1.1 (6)
C14—N3—C1—N2	59.1 (4)	C3—C5—C9—S1	0.3 (4)
C2—N2—C1—N1	0.9 (5)	C6—C5—C9—S1	178.6 (3)
C15—N2—C1—N1	−167.9 (3)	S2—C8—C9—C5	−18.4 (5)
C2—N2—C1—N3	177.6 (3)	S2—C8—C9—S1	164.27 (19)
C15—N2—C1—N3	8.9 (4)	C4—S1—C9—C5	−0.5 (3)
C1—N2—C2—O1	179.9 (3)	C4—S1—C9—C8	177.2 (3)
C15—N2—C2—O1	−10.6 (4)	C1—N3—C10—C11	153.3 (3)
C1—N2—C2—C3	−0.7 (4)	C14—N3—C10—C11	−60.0 (4)
C15—N2—C2—C3	168.7 (3)	N3—C10—C11—C12	56.0 (5)
O1—C2—C3—C4	178.4 (3)	C10—C11—C12—C13	−53.2 (5)
N2—C2—C3—C4	−0.9 (4)	C11—C12—C13—C14	52.7 (5)
O1—C2—C3—C5	1.4 (5)	C1—N3—C14—C13	−153.0 (3)
N2—C2—C3—C5	−177.9 (3)	C10—N3—C14—C13	61.5 (4)
C1—N1—C4—C3	−2.5 (5)	C12—C13—C14—N3	−56.6 (4)
C1—N1—C4—S1	178.2 (2)	C1—N2—C15—C16	62.8 (4)
C2—C3—C4—N1	2.7 (5)	C2—N2—C15—C16	−106.7 (3)
C5—C3—C4—N1	−179.9 (3)	C1—N2—C15—C20	−119.3 (3)
C2—C3—C4—S1	−177.9 (2)	C2—N2—C15—C20	71.1 (4)
C5—C3—C4—S1	−0.5 (4)	C20—C15—C16—C17	−0.2 (5)
C9—S1—C4—N1	−180.0 (3)	N2—C15—C16—C17	177.6 (3)
C9—S1—C4—C3	0.6 (3)	C15—C16—C17—C18	−0.3 (5)
C4—C3—C5—C9	0.1 (4)	C16—C17—C18—C19	0.8 (6)
C2—C3—C5—C9	177.2 (3)	C17—C18—C19—C20	−0.9 (6)
C4—C3—C5—C6	−178.2 (3)	C16—C15—C20—C19	0.1 (5)
C2—C3—C5—C6	−1.1 (5)	N2—C15—C20—C19	−177.7 (3)
C9—C5—C6—C7	−18.4 (5)	C18—C19—C20—C15	0.4 (6)
C3—C5—C6—C7	159.7 (3)		

Symmetry codes: (i) −*x*, −*y*+1, −*z*−1.

### Hydrogen-bond geometry (Å, °)

**Table:**

*D*—H···*A*	*D*—H	H···*A*	*D*···*A*	*D*—H···*A*
C7—H7B···O1^ii^	0.97	2.56	3.321 (5)	136

Symmetry codes: (ii) −*x*+1, −*y*+1, −*z*.
